# Molecular characterization of *Neisseria meningitidis* isolates recovered from 11-19-year-old meningococcal carriers in Salvador, Brazil

**DOI:** 10.1371/journal.pone.0185038

**Published:** 2017-09-20

**Authors:** Ana Rafaela Silva Simões Moura, Cécilia Batmalle Kretz, Italo Eustáquio Ferreira, Amélia Maria Pithon Borges Nunes, José Cássio de Moraes, Mitermayer Galvão Reis, Alan John Alexander McBride, Xin Wang, Leila Carvalho Campos

**Affiliations:** 1 Laboratório de Patologia e Biologia Molecular, Instituto Gonçalo Moniz, FIOCRUZ-BA, Salvador, Bahia, Brazil; 2 Meningitis and Vaccine Preventable Diseases Branch, Division of Bacterial Diseases, Centers for Disease Control and Prevention, Atlanta, United States of America; 3 Faculdade de Ciências Médicas da Santa Casa de São Paulo, São Paulo, Brazil; 4 Núcleo de Biotecnologia, Centro de Desenvolvimento Tecnológico, Universidade Federal de Pelotas, Pelotas, Rio Grande do Sul, Brazil; Universidad Nacional de la Plata, ARGENTINA

## Abstract

Characterization of meningococci isolated from the pharynx is essential towards understanding the dynamics of meningococcal carriage and disease. Meningococcal isolates, collected from adolescents resident in Salvador, Brazil during 2014, were characterized by multilocus sequence typing, genotyping or whole-genome sequencing. Most were nongroupable (61.0%), followed by genogroups B (11.9%) and Y (8.5%). We identified 34 different sequence types (STs), eight were new STs, distributed among 14 clonal complexes (cc), cc1136 represented 20.3% of the nongroupable isolates. The *porA* and *fetA* genotypes included P1.18,25–37 (11.9%), P1.18–1,3 (10.2%); F5-5 (23.7%), F4-66 (16.9%) and F1-7 (13.6%). The *porB* class 3 protein and the *fHbp* subfamily A (variants 2 and 3) genotypes were found in 93.0 and 71.0% of the isolates, respectively. NHBA was present in all isolates, and while most lacked NadA (94.9%), we detected the hyperinvasive lineages B:P1.19,15:F5-1:ST-639 (cc32); C:P1.22,14–6:F3-9:ST-3780 (cc103) and W:P1.5,2:F1-1:ST-11 (cc11). This is the first report on the genetic diversity and vaccine antigen prevalence among *N*. *meningitidis* carriage isolates in the Northeast of Brazil. This study highlights the need for ongoing characterization of meningococcal isolates following the introduction of vaccines and for determining public health intervention strategies.

## Introduction

*Neisseria meningitidis* is a human commensal bacterium that commonly colonizes the oropharyngeal mucosa, occasionally causing life-threatening disease, such as meningitis or septicemia [1] Meningococcal populations possess a diverse and dynamic structure [2,3]. However, most invasive meningococcal cases are caused by a limited number of clonal complexes (cc), known as hyperinvasive lineages, which persistently exist over time despite high rates of recombination [3,4]. The population structure of meningococcal carriage strains is less well defined [5] and some are associated with hypervirulent lineages [6]. Most carriage meningococci lack a capsule and are thus nongroupable (NG). However, commensal strains may play an important role as a reservoir of virulence genes, with implications for meningococcal diversity due to the high frequency of recombination [3].

Multilocus sequence typing (MLST) is used for studying population biology and the evolution of microorganisms [4] and the PubMLST database allows the comparison of global meningococcal strains [7]. While MLST has a low discriminatory power, this has been overcome by characterizing the genes encoding several outer membrane proteins, including: porins A (PorA) and B (PorB) and iron-regulated enterobactin (FetA) [8]. Typing of factor H-binding protein (FHbp), Neisserial adhesion A (NadA) and Neisserial heparin binding antigen (NHBA) can also improve meningococcal typing and provide information on strain coverage conferred by the serogroup B meningococcal (MenB) vaccines [9]. These antigens were used in the development of two MenB vaccines, the MenB-4C multi-component recombinant vaccine and the MenB-FHbp bivalent vaccine [9, 10].

In Brazil, meningococcal disease is endemic with an annual incidence of 1.5–2.0 cases per 100,000 inhabitants [11]. Serogroup C has been responsible for most cases and is historically associated with ST-11 during the 1970s and ST-103 after 2000 [12]. However, there is only limited data describing meningococcal carriage in Brazil [13,14].

Characterization of meningococci isolated from the pharynx is essential towards understanding the dynamics of meningococcal carriage and disease and to determine the potential impact of disease control programs, such as vaccination, on the transmission of meningococci. In 2014, we conducted a cross-sectional study to assess the meningococcal carriage status of 11-19-year-old student’s resident in Salvador [15]. In the current work, the meningococcal carriage isolates were characterized by capsular group, ST, and the presence and sequence variability of the *por*A, *por*B, *fet*A, *fHbp*, *nhba*, and *nadA* genes.

## Materials and methods

### Ethics statement

This study was approved by the Ethics Committee at the Gonçalo Moniz Institute, FIOCRUZ-BA (CAEE # 16099713.1.0000.0040). Written informed consent from all participants (or guardians) in the study were obtained before sample and data collection.

### Meningococcal isolates

Meningococcal isolates (*n* = 59) were recovered from oropharyngeal swabs collected from 1,200 students, aged 11–19 years old, attending 134 public schools in Salvador, Brazil, during September-December 2014. Some 59 participants (4.9%) were found to be meningococcal carriers as described previously [15]. The swab was immediately plated onto a selective agar medium (modified Thayer-Martin vancomycin, colistin, nystatin, and trimethoprim) and introduced in plastic tubes containing 1 mL of skim milk-tryptone-glucose-glycerin (STGG) transport medium[16]. Meningococcal identification was determined by Gram staining (BD BBL, Sparks, MD), the oxidase reaction (BD BBL Dryslide, Cockeysville, MD), and carbohydrate utilization tests. Results were confirmed by API-NH^®^ strips (bioMérieux, Hazelwood, MO). The isolates were stored at −80°C in brain heart broth with 20% glycerol.

### Capsular typing

Capsular groups were characterized by real-time PCR (qPCR), the primers and probes for the *ctrA* and *sodC* genes and for serogroups A, B, C, W, Y and X were used as described previously [17,18],. The capsule null locus (*cnl*) was detected by PCR amplification and sequencing as described previously [19].

### Multilocus sequence typing (MLST)

MLST was performed according to the method described by Maiden et al. [4]. STs and cc were assigned by searching the *Neisseria* PubMLST database (http://pubmlst.org/neisseria/). Sequence data were assembled and alleles were determined using the Meningococcus Genome Informatic Platform (MGIP, http://mgip.biology.gatech.edu) or SeqMan Pro, ver12.2 (DNASTAR, Inc.).

### Outer membrane protein typing

The amplification and sequencing of the *porA*, *porB*, *fetA*, *fHbp*, *nhba* and *nadA* genes were performed as previously described [20–23]. Alleles and protein variants were assigned using the *Neisseria* PubMLST database.

### Whole-genome sequencing

The *N*. *meningitidis* isolates that were not fully characterized by molecular typing were analyzed by whole-genome sequencing. Genomic DNA was extracted [24] and sequenced using MiSeq v2 chemistry (Illumina, San Diego, CA, USA). Genome assembly was carried out using CLC Genomics Workbench, ver 9.0.0 (CLC bio, Aaarhus, Denmark) with read trimming and mapping of reads back to contigs. The MLST alleles, STs and cc were identified by comparison of the assembled genomes with PubMLST [7] alleles using a BLAST search (https://blast.ncbi.nlm.nih.gov/Blast.cgi). Sequences of PorA, PorB, FetA, NadA, NHBA and FHbp were identified as described previously [24].

### Phylogenetic analysis

Single nucleotide polymorphisms (SNPs) were identified using kSNP version 3 software [25] with a kmer length of 25. A maximum likelihood phylogenetic tree was constructed from the core SNPs and the Tamura-Nei model, using MEGA7 [26] and 500 bootstraps iterations.

## Results

### Capsular typing

Of the 59 *N*. *meningitidis* isolates analyzed, 61.0% (36/59) were NG, and 50.0% (18/36) lacked capsular genes (capsule null). Most groupable isolates belonged to genogroup B (7/59; 11.9%), followed by Y (5/59; 8.5%), E (4/59; 6.8%), Z (3/59; 5.1%), C (2/59; 3.4%) and W (2/59; 3.4%). None of the study participants were colonized by either *N*. *meningitidis* genogroup A or genogroup X.

### MLST profiles

Thirty-four different STs were identified, eight (23.5%) of which were described for the first time in this study and were registered in the PubMLST database (Table 1). Overall, 83.1% (49/59) of the isolates fell into 14 known cc. The most frequent were cc1136 (*n* = 12; 20.3%) and cc198 (*n* = 11; 18.6%), in NG strains. Hyperinvasive lineage complexes were also detected and included: cc23 (*n* = 4; 6.8%); cc41/44 (*n* = 3; 5.1%); cc32 (*n* = 2; 3.4%), cc11 (*n* = 2; 3.4%), cc35 (*n* = 1; 1.7%), cc103 (*n* = 1; 1.7%) and cc175 (*n* = 1; 1.7%) (Table 1).

**Table 1:** Genotypic characterization of the 59 *N*. *men*ingitidis isolates.

Capsular Type	ST	Clonal Complex	PorA	PorB	FetA	FHbp†	NHBA‡	NadA	N° of Isolates
B	639	32	P1.19,15	3–1	F5-1	2.19	231	1.1	1
	2120	41/44	P1.18–7,9	3–16	F1-5	2.19	2	No	1
	**11453**	NA	P.1.19,15–1	3–45	F1-7	2.24	21	No	1
	3200	4821	P1.17–6,23–6	3–372	F3-36	2.16	669	No	1
	3496	213	P1.22,14–26	3–14	F5-9	3.45	18	4/5	1
	**11577**	41/44	P1.22,14–6	3–64	F1-199	2.19	291	No	1
	5892	41/44	P1.7–2,13–1	3–66	F4-38	2.19	335	No	1
C	3771	35	P1.7–2,13–7	3–154	F1-7	2.24	21	No	1
	3780	103	P1.22,14–6	2–23	F3-9	2.25	24	No	1
W	11	11	P1.5,2	2–3	F1-1	2.151	29	2/3	1
	7097	11	P1.5,2	2–3	F1-1	2.151	29	2/3	1
Y	**11545**	NA	P1.5–1,10–4	3–100	F1-7	2.21	9	No	1
	23	23	P1.5–2,10–2	3–53	F1-3	2.104	8	No	1
	**11452**	NA	P1.18–1,3	2–194	F3-4	2.16	20	No	1
	**11461**	23	P1.7–2,¶	3–36	F1-5	2.21	145	No	1
	5770	175	P1.5–1,10–3	3–100	F1-7	2.21	9	No	1
E	10220	254	P1.7–1,1	3–38	F3-67	1.13	9	No	2
	10220	254	P1.7–1,1	3–38	F3-67	2.104	9	No	1
	10224	254	P1.21,16	3–320	F3-9	1.13	9	No	1
Z	**11458**	NA	P.19,15	3–1	F5-7	2.16	101	2/3.8	1
	2123	NA	P1.18–1,3	3–38	F5-7	2.25	101	No	1
	5953	NA	P1.18–1,30–2	3–63	F5-7	2.22	92	No	1
NG	823	198	P1.18,25–37	3–84	F5-5	1.4	10	No	3
	6525	NA	P1.5–11,10–13	3–100	F1-7	2.21	101	No	2
	1136	1136	P1.18–1,3–4	3–84	F4-66	3.94	600	No	2
	1136	1136	P1.18–1,3	3–84	F4-66	3.94	600	No	1
	4210	178	P1.19–5,15–23	3–38	F1-7	1.12	6	No	1
	823	198	P1.18,25–37	3–84	F5-5	1.1	10	No	1
	823	198	P1.18,25–44	3–84	F5-5	1.4	10	No	1
	823	198	P1.18–1,30–4	3–381	F5-5	1.4	10	No	1
	1136	1136	P1.18–1,3–8	3–84	F4-66	3.31	600	No	1
	6519	23	P1.18–1,30–2	3–36	F5-5	2.21	145	No	1
	6519	23	P1.18–1,30	3–36	F5-5	2.21	145	No	1
	6525	NA	P1.5–11,10–13	3–100	F1-7	2.21	9	No	1
	**11451**	198	P1.18,25–37	3–84	F5-5	1.4	10	No	1
	639	32	P1.19,15	3–1	F5-1	2.19	145	1.1	1
*cnl*§	1136	1136	P1.18–1,3	3–84	F4-66	3.94	600	No	3
	823	198	P1.18,25–37	3–84	F5-5	1.4	10	No	2
	53	53	P1.7,30–3	3–64	F1-2	2.102	58	No	2
	53	53	P1.7–2,30–3	3–64	F1-5	2.102	58	No	1
	53	53	P1.7,30–2	3–64	F1-5	2.102	65	No	1
	53	53	P1.7,30–45	3–64	F1-2	2.102	58	No	1
	823	198	P1.18,25–32	3–37	F5-5	1.4	10	No	1
	1136	1136	P1.18–1,3–7	3–84	F4-66	3.94	600	No	1
	7129	NA	P1.12–6,13–39	3–122	F5-5	3.627	308	No	1
	7450	1136	P1.18,25	3–37	F2-7	3.94	145	No	1
	**11459**	1136	P1.18–4,25–60	3–84	−¶¶	3.31	262	No	1
	**11462**	198	P1.22–1,14–23	3–84	F5-5	1.4	10	No	1
	10238	1136	P1.18–4,25–50	3–84	F4-66	3.94	600	No	1
	**11552**	1136	P.18-1,3–8	3–84	F4-66	3.94	600	No	1

* ST, sequence type; PorA, porin A; PorB, porin B; FetA, iron-regulated enterobactin; FHbp, factor H binding protein; NHBA, neisserial heparin binding antigen; NadA, *Neisseria* adhesion A; NA, clonal complex not assigned; NG, non genogroupable. The new STs that were described for the first time in this study are indicated in bold.

^†^Nomenclature using variant peptide subvariant.

^‡^Novartis nomenclature.

^¶^PorA VR2 variant was not found.

^§^*cnl*, Capsule null locus.

^¶¶^*fetA* gene deleted.

**Fig 1:**
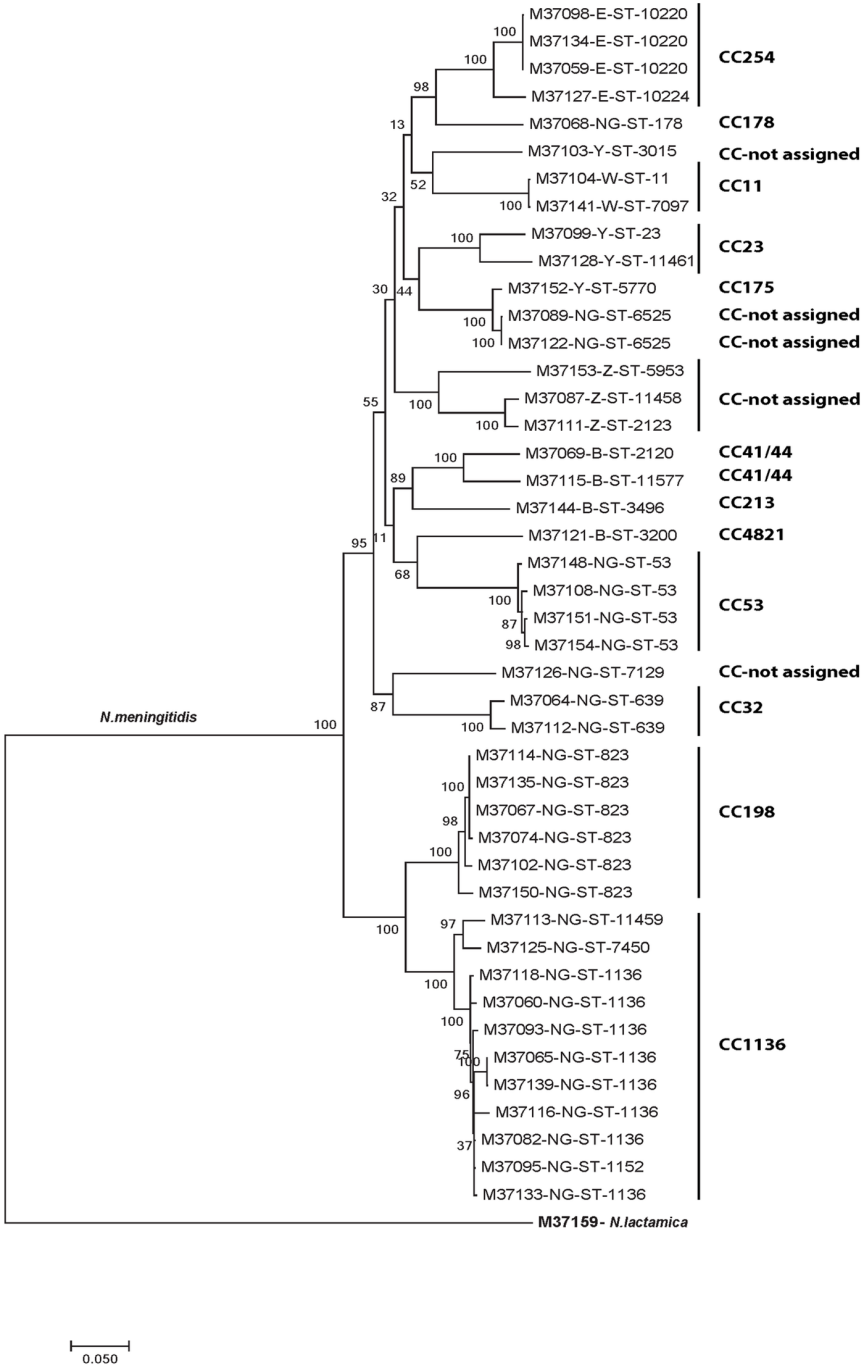
Phylogenetic tree of the *N*. *meningitidis* isolates based on the whole-genome sequence data. The *N*. *meningitidis* isolates are labelled with their sample ID, serogroup (SG), sequence type (ST) and clonal complex (cc). An *N*. *lactamica* isolate was used as the outgroup. Internal nodes are labeled with bootstrap values. The scale bar is based on the 7131 positions in the core SNP matrix and indicated nucleotide substitutions per site.

An association between the capsular groups and cc was observed: cc1136, cc198 and cc53 were associated with NG strains; cc32 and cc41/44 were found among serogroup B isolates; cc103 was related to serogroup C strains, and cc11 was found among serogroup W isolates (Fig 1). Furthermore, three NG strains were associated with the hypervirulent cc23 (*n* = 2) and cc32 (*n* = 1).

Using the European Meningococcal Disease Society (EMGM) recommended strain designation for meningococci [8], we identified 44 finetypes including: NG:P1.18,25–37:F5-5:ST-823 (cc198) (*n* = 7; 11.9%) and NG:P1.7–1,1:F4-66:ST-1136 (cc1136) (*n* = 4; 6.8%). We also found isolates belonging to the hyperinvasive lineages W:P1.5,2:F1-1:ST-11 (cc11) (*n* = 1), B:P1.19,15:F5-1:ST-639 (cc32) (*n* = 1), C:P1.22,14–6:F3-9:ST-3780 (cc103) (*n* = 1) (Table 1).

### Outer membrane protein typing

The *porA*, *porB*, *fetA*, *fHbp*, *nhba*, and *nadA* genes were characterized in all 59 isolates (Table 1). A total of 36 different PorA types (P1.VR1,VR2) were identified, including 18 VR1 variants and 34 VR2 variants. The most common PorA was P1.18,25–37 (*n* = 7, 11.9%), followed by P1.18–1,3 (*n* = 6, 10.2%). None of the isolates contained the VR2 variant 4 present in the MenB-4C vaccine and only one serogroup Y (cc23) isolate contained the VR2 variant 4 (Table 1). We found a predominance of PorB class 3 proteins (*n* = 55, 93.2%), and PorB 3–84 (*n* = 20, 33.9%) was the most prevalent. We also identified five novel PorB genotypes: 2–194, 3–36, 3–122, 3–320, and 3–381 (Table 1).

Among the 17 FetA variants identified, the most frequent was F5-5 (*n* = 14, 23.7%), followed by F4-66 (*n* = 10, 16.9%) and F1-7 (*n* = 8, 13.6%). The *fetA* gene was deleted in only one NG isolate belonging to cc1136; five isolates belonging to different cc included FetA variants that are associated with hypervirulent lineages, including F5-1 (*n* = 2), F3-9 (*n* = 2), and F2-7 (*n* = 1) [27].

All three variants (two subfamilies) of the vaccine antigen FHbp were identified, the v2 variant was the most prevalent (*n* = 30; 50.8%), followed by v1 (*n* = 15; 25.4%) and v3 (*n* = 14; 23.7%). Overall, the most prevalent FHbp subvariants were: FHbp-3.94 (*n* = 10; 16.9%) and FHbp-1.4 (*n* = 10; 16.9%), associated with cc1136 and cc198, respectively; FHbp-2.21 (*n* = 8; 13.6%), associated with cc23 (3 isolates), cc175 (1 isolate), or were not assigned to any cc (4 isolates). FHbp-1.1, present in the MenB-4C vaccine [28], was found in only one isolate associated with cc198 (Table 1).

We identified 20 unique NHBA subvariants: NHBA-10 was the most frequent (*n* = 11, 18.6%), associated with cc198, followed by NHBA-600 (*n* = 10, 16.9%), associated with cc1136 (Table 1). Only one isolate (genogroup B; cc41/44) contained the NHBA-2 variant that is included in the MenB-4C vaccine. Most of the isolates lacked *nadA* (*n* = 56, 94.9%), and none of the isolates included the NadA-3 variant present in the MenB-4C vaccine [28].

### Genomic diversity of the *N*. *meningitidis* isolates

The genetic relatedness of the 45 *N*. *meningitidis* isolates that could not be fully characterized by molecular typing was assessed using whole-genome sequencing. The phylogenetic analysis revealed that isolates from the same cc clustered together (Fig 1). A total of 7131 core SNPs were identified with a difference of 0–3847 between all isolates analyzed.

## Discussion

In the present study, we evaluated the molecular characteristics of meningococcal carriage isolates recovered from 11-19-year-old students, resident in Salvador, Brazil. Most of the *N*. *meningitidis* isolates were NG, which is consistent with other carriage studies [19,28,29]. Although the capsule is not required for person-to-person transmission [19] there is evidence that loss of the capsule enhances the capacity of meningococci to colonize the human nasopharynx and to avoid human defense systems [30]. Furthermore, in some instances, capsule-deficient strains have caused invasive disease [31].

Among the groupable carriage isolates, the most common included genogroups MenB and MenY, in agreement with previous reports [28,32,33]. In addition, we found a low prevalence of MenC carriage among the students, which may be related to the mass vaccination campaign with a MenC conjugate vaccine that was conducted in Salvador in 2010 [34]. Although the vaccination status of the participants was not available, the MenC vaccination campaign for 10-24-year-olds may have had some effect on the low MenC colonization rates seen in this study. As seen in studies from the United Kingdom, the introduction of a MenC conjugate vaccine to the adolescent and young adult population was responsible for a 67% reduction in MenC colonization rates compared to non-vaccinated individuals [35]. However, we were unable to evaluate the impact of MenC conjugate vaccine on meningococcal carriage due to the lack of baseline carriage data prior to the vaccination campaign.

Molecular typing revealed that the *N*. *meningitidis* isolates were highly diverse, as expected for a carrier population [2]. We characterized 34 STs belonging to 14 cc and found an association with some of the capsular groups, as previously reported [29,36]. The cc1136 and cc198 were most common and, as observed in our study, these cc can be found among carriage and *cnl*-positive isolates [36]. Indeed, the genetic relatedness of the 45 isolates analyzed by whole-genome sequencing found that isolates belonging to the same cc were more closely related and formed distinct phylogenetic clusters (Fig 1).

In agreement with previous reports of carriage and invasive isolates, we found an association between genogroup B and cc41/44, cc32 and cc4821 [6,28,33]. Interestingly, one of the NG cc32 isolates clustered with a genogroup B cc32 isolate and had the same genotype profile except for the NHBA protein variant (Table 1). This NG cc32 isolate lacked the *csb* gene, which is required for capsule synthesis.

Genogroup Y was associated with cc23 and cc175 and isolates belonging to cc23 have been reported to be involved with invasive disease in the USA, South America, Europe and South Africa [37]. Furthermore, cc175 was responsible for over 17% of MenY invasive cases in Brazil, during 2007–2011 [38]. These results demonstrate the continuing circulation of pathogenic isolates among carriers.

Previous studies found that a small proportion of carriage isolates belonged to hyperinvasive lineages [6,29]. Furthermore, it is known that these lineages can persist over many decades and spread around the world, despite high rates of recombination [3]. In this study, we identified three hyperinvasive isolates associated with meningococcal disease cases in Brazil. Of note, the strain C:P1.22,14–6:F3-9:ST-3780 (cc103), differing only in the PorA VR1 subtype, is responsible for most meningococcal disease cases in Brazil. It was also identified as the causative agent of the last outbreak that occurred in Bahia State, Brazil, in 2010 [34,39]. The MenB hyperinvasive isolate B: P1.19,15: F5-1: ST-639 (cc32) has been found in almost all Brazilian states, with the highest prevalence in the Northeast region [40]. Similarly, the genogroup W isolate, with the profile W: P1.5,2: F1-1: ST-11 (cc11), has been linked to an increase in endemic meningococcal disease in many regions, including England, South Africa and South America countries, including Brazil, where case fatality rates reached 28% [41–43]. There has been an increase in the number of meningococcal disease cases associated with MenW in South America, including Brazil, where this is now the third most prevalent serogroup [41,44]. Such findings show the need for continuous surveillance, not only phenotypically but including the molecular characterization of the strains, due to the high transmissibility and virulence of the circulating genotype.

There are few reports describing the distribution of the vaccine antigen alleles among *N*. *meningitidis* carriage isolates [28,45]. Overall, the PorA, PorB and FetA variants identified in this study were highly variable within the same genogroup and cc, as well as the presence of the same antigenic allele in different cc.

This study showed that almost 95% of the isolates lacked NadA, which confirmed the observation that only approximately 5% of the carrier population harbor strains with this protein [46]. In addition, we found an association between NadA and some of the hypervirulent cc including: NadA-1 (cc32); NadA-4 (cc2132) and NadA-2 (cc11) [45,46]. Furthermore, we observed associations such as FHbp 2.102, NHBA 58 and PorB 3–64 variants among *cnl* (cc53) strains; FHbp 1.4, NHBA 10 and PorB 3–84 among *cnl* (cc198) strains; FHbp 3.94, NHBA 600 and PorB 3–84 among *cnl* (cc1136) strains; and NHBA 21 and NHBA 24 associated with MenC cc35 and cc103, respectively, as previously reported [28,45,47].

Considering the MenB-4C vaccine components globally, the NHBA-2 and FHbp 1.1 variants were found in carriage isolates B: P1.18–7.9: F1-5: ST-2120 (cc41/44) and NG: P1.18.25–37: F5-5: ST-823 (cc198), respectively. Some studies reported high cross-reactivity among homologous FHbp-1 subvariants, heterologous NHBA subvariants, and among NadA-1, NadA-2 and NadA-3 variants [48,49]. Moreover, studies on the effectiveness of the MenB-4C vaccine have shown that the presence of at least one of the components may be able to induce protection against both genogroup B and non-genogroup B isolates [50].

In conclusion, this study presents an overview of the molecular diversity and vaccine antigen content of *N*. *meningitidis* carriage isolates in Salvador, Brazil. Continuous monitoring of antigen variability, including carriage isolates from other age groups, as well as isolates from meningococcal cases, will be needed to monitor the impact of the anti-meningococcal vaccination strategies on the carriage population, as well as to contribute to future public health decisions on vaccine usage.

## Supporting information

S1 Text Database. (XLSX)
